# Supplementary material for: A phenomenological exploration of mental toughness in decision-making: Perceptions from professional Ghanaian footballers
Source: PLoS One. 2026 Feb 12;21(2):e0342778. doi: 10.1371/journal.pone.0342778 (PMC12900318; doi:10.1371/journal.pone.0342778)
Supplement: S1 Questionnaire — (DOCX) [file pone.0342778.s001.docx]

Inclusivity in global research

PLOS’ policy on inclusivity in global research aims to improve transparency in the reporting of research performed outside of researchers’ own country or community and ensures that PLOS publications reporting global research adhere to high standards for research ethics and authorship. Authors of relevant research articles may be asked to complete the questionnaire below, which outlines ethical, cultural, and scientific considerations specific to inclusivity in global research. This questionnaire may be requested when researchers have travelled to a different country to conduct research, if research uses samples collected in another country, research with Indigenous populations or their lands, or if research is on cultural artefacts. Researchers travelling to another country solely to use laboratory equipment will not normally be required to complete the questionnaire. However, the questionnaire can be requested at the journal’s discretion for any submission – if you have been requested to complete this questionnaire by the PLOS journal you submitted to, please do so.

Please complete the questionnaire below and include this as a Supporting Information file with your manuscript. Note that if your paper is accepted for publication, this checklist will be published with your article in the supporting information files. Please ensure that you reference the checklist in the main body of your manuscript. We suggest adding a subsection ‘Inclusivity in global research’ to your Methods section and adding the following sentence: “Additional information regarding the ethical, cultural, and scientific considerations specific to inclusivity in global research is included in the Supporting Information (SX Checklist)”

The questions have been designed to be applicable to a wide range of study types, and there are subsections for both human subjects research and non-human subjects research. If any of the questions are not relevant to your research please mark them as “N/A” as appropriate.

**Ethical considerations, permits and authorship**

*This section is applicable to all research types.*

Provide details as to who granted permissions and/or consent for the study to take place in the Methods section of your manuscript. This should include the names of **all** ethics boards, governmental organizations, community leaders or other bodies that provided approval for the study. If individuals provided approval refer to these people by their role or title but do not list their name(s).

Ethical approval for this study was obtained from the Stellenbosch University Research Ethics Committee for Social, Behavioural, and Educational Research (REC: SBE – 22265) and the Kwame Nkrumah University of Science and Technology Committee on Human Research, Publication and Ethics (CHRPE/AP/304/21). Permission to use the Ghana Premier League platform for participant recruitment was granted by the Ghana Football Association. Additionally, all individual participants (professional footballers) provided written informed consent for their inclusion in the study. Complete details regarding ethical approvals and consent procedures are provided in the Ethical considerations subsection of the Methods section on page 8 of the manuscript.

If there were any deviations from the study protocol after approval was obtained please provide details of these changes in the Methods section of your manuscript.
Did this study involve local collaborators that are residents of the country where the research was conducted or members of the community studied? If you do not have any authors from said communities, please provide an explanation for this below.

Yes. The First author and second author (PhD co-supervisor) are both indigenous Ghanaians and residetnts of Ghana, affiliated with the Kwame Nkrumah University of Science and Technology (KNUST), Kumasi, Ghana. The first author is a PhD candidate registered at Stellenbosch University, South Africa, where the third author serves as his doctoral supervisor. All data collection, transcription, and manuscript preparation which forms part of the first author’s PhD dissertation were conducted in Ghana. This collaborative structure ensured the research was designed, implemented, and analysed by local researchers who are members of the Ghanaian academic and sporting community where the study was conducted, with international supervisory support for doctoral training purposes.

The study did not deviate from the study protocol after ethical approval was obtained. All research activities were conducted in accordance with the approved protocol and procedures

Everyone listed as an author should meet PLOS’ criteria for authorship and all individuals who meet these criteria should be included in the author byline, rather than the acknowledgements. For further information please see the journal’s Authorship Policy.

**Human subjects research (e.g. health research, medical research, cross-cultural psychology)**

Did you obtain written informed consent from a representative of the local community or region before the research took place? How did you establish who speaks for the community? Details of written informed consent obtained from study participants should be reported separately in the Methods section of your manuscript.

Yes. The authors obtained permission from the Ghana Football Association (GFA), the authorised governing body for all football-related matters in Ghana, to use their platform (Ghana Premier League) for participant recruitment. The GFA is the recognized national authority that speaks for and regulates the professional football community in Ghana.Prior to data collection, the lead author engaged the GFA to discuss the study objectives, data collection procedures, and anticipated benefits. Following these consultations, an official letter was written to the GFA requesting formal approval for the study. The GFA granted permission and appointed a technical officer to serve as liaison to facilitate access to potential participants for the study. Additionally, written informed consent was obtained from all study participants before commencement of data collection. All participants were assured that participation was voluntary and they could withdraw from the study at any time without prejudice. Confidentiality and anonymity of study participants were also assured. Complete details regarding informed consent process and ethical procedures are provided in the Ethical consideration subsection of the methods section on page 8 of the manuscript.

How did members of the local community provide input on the aims of the research investigation, its methodology, and its anticipated outcome(s)?

Local community input was integrated throughout the research design process. Initial consultations between the first author and the technical directorate of the Ghana Football Association provided critical insights into how to appropriately approach potential participants, given that the Ghanaian football ecosystem is not always embracing of reseatchers intriduing into their space. These consultations informed culturally appropriate recruitment strategies and engagement approaches. Additionally, the first author engaged an expert with extensive technical experience within the Ghanaian football ecosystem to review the interview guide for sociocultural appropriateness and contextual relevance. This consultation resulted in the reordering of questions to enhance cultural sensitivity and optimize participant engagement. Furthermore, the interview guide was pretested with three professional players to ascertain whether the questions adequately addressed the study objectives, assess the conceptual flow of questions, and identify any practical concerns. Feedback from this pretesting process was incorporated to refine the interview guide before commencing formal data collection. This collaborative approach ensured the research aims, methodology, and procedures resonated with local stakeholders and reflected the cultural realities of the Ghanaian football community.

When engaging with the local community, how did you ensure that the informed consent documents and other materials could be understood by local stakeholders?

Multiple strategies were employed to ensure information consent documents and study materials were fully understood by local staksholders. First, the informed consent documents and interview guide were vetted by the Ghana Football Association to ensure appropriateness for the professional football community. Second, all material were translated from English to Twi (local vernacular) to ensure accessibility and comprehension.

The first author, who is fluent in both English and Twi, conducted all face-to-face interviews in either English or Twi according to participant preferences and the language in which they are most comfortable, in accordance with ethical practices. Prospective participants were fully briefed about the study aims, potential benefits and risks, the fact that interviews would be audio-recorded, confidentiality and anonymity protocols, their rights as participants, and their liberty to withdraw from the study at any time without prejudice, as reflected in the informed consent documents. All participants in the study were literate. The first author provided informed consent forms to prospective participants to read for themselves and ask any questions before providing consent. Interviews were scheduled one week from first contact with prospective participants, allowing adequate time for participants to review materials, consider their participation, and seek clarification on any concerns without pressure. This multi-layered approach ensured that all materials were fully understandable and that participants could make truly informed voluntary decisions about participation.

Will the findings of the research be made available in an understandable format to stakeholders in the community where the study was conducted (e.g. via a presentation, summary report, copies of publications, etc.)? Please provide details of how this will be achieved.

Yes. The findings will be disserminated to the Ghanaian football community through multiple channels to ensure accessibility and practical application. First, a plain-language summary report highlighting key findings and practical implications will be prepared. This report will be written in non-technical language accessible to football practioners and shared with the technical directorate of the Ghana Football Association for distribution to clubs, coaches, and players during their technical meetings. Where necessary, brief explanatory notes will be included to contextualise the findings within everyday football practices, ensuring stakeholders can readily interpret and apply the insights.

Second, findings will be presented in stakeholder-focused forums, such as coaches’ workshops, technical meetings, or professional development sessions organised by the Ghana Football Association or affiliated bodies. These presentations will emphasise applied insights related to decision-making across temporal phases of players’ careers, allowing stakeholders to engage with the findings in an interactive and dialogical manner. This format is facilitates discussion, questions, and practical application within the Ghanaian football context.

Third, copies of peer-reviewed publications arising from this research will be shared with relevant institutional stakeholders, including the Ghana Football Association, participating clubs, and academic departments involved in sports science and coaching education. This multi-pronged dissermination strategy ensures that research benefits return to the community that enabled the investigation and that findings inform both practice and policy within Ghanaian football.

**Non-human subjects research using specimens/ animals collected as part of the study, or those housed in archival collections. Examples include archaeology, paleontology, botany and zoology.**

Did the permission you obtained from a local authority to perform the study include an agreement on access to outputs and benefit sharing? This may include procedures to enable fair distribution of the benefits and resources arising from the research performed. Please include any details of Prior Informed Consent and Benefit Sharing Agreements obtained. These may be required by field-specific regulations, for example the Convention on Biological Diversity (CBD) and the associated Nagoya Protocol.

N/A

If the material used in your study was imported, please A) provide the year it was imported and B) indicate whether permits were obtained to import/export the materials used, C) provide details of any permits obtained. If this information is not available, please indicate this.

N/A

If you used archival specimens, please state how the material used in your study was acquired by the institute it is held in and provide details of any permits obtained for the original excavations/ sample collection. If this information is not available, please indicate this.

N/A

How was the potential cultural significance of the materials collected in your study to local communities considered in your research design? Were Indigenous peoples and/or local researchers and institutions involved with archaeological excavations / collection of specimens? If so, please provide a description of their involvement.

N/A

If your manuscript includes photographs of human remains please indicate whether authors obtained permission from descendants or affiliated cultural communities to do so.

N/A
