# Supplementary material for: A phenomenological exploration of mental toughness in decision-making: Perceptions from professional Ghanaian footballers
Source: PLoS One. 2026 Feb 12;21(2):e0342778. doi: 10.1371/journal.pone.0342778 (PMC12900318; doi:10.1371/journal.pone.0342778)
Supplement: Attachment — Submitted filename: Response to Reviewers.docx [file pone.0342778.s003.docx]

08/12/2025

Dear guest editor (Wanli Zang) and reviewers

**Authors’ response to the review of Manuscript PONE-D-25-33728**

**“A Phenomenological Exploration of Mental Toughness in Decision-making: Perceptions from Professional Ghanaian Footballers"**

Our sincere appreciation for your thorough review, encouraging feedback and constructive suggestions. Please see our response(s) to each of the comments/recommendations below. We used track changes to revise our manuscript.

1. Please ensure that your manuscript meets PLOS ONE's style requirements, including those for file naming. The PLOS ONE style templates can be found at

<https://journals.plos.org/plosone/s/file?id=wjVg/PLOSOne_formatting_sample_main_body.pdf> and

[https://journals.plos.org/plosone/s/file?id=ba62/PLOSOne_formatting_sample_title_authors_affiliations.pdf](https://zaf01.safelinks.protection.outlook.com/?url=https%3A%2F%2Ftrack.editorialmanager.com%2FCL0%2Fhttps%3A%252F%252Fjournals.plos.org%252Fplosone%252Fs%252Ffile%253Fid%3Dba62%252FPLOSOne_formatting_sample_title_authors_affiliations.pdf%2F1%2F010f019a28db5b1b-094911dd-37d9-4638-8179-149c73c480e6-000000%2Fni6SDNgdO048-eLzv5dEyAUkiMJk0kSkmsflTkbUnCY%3D234&data=05%7C02%7C%7C4bb682144a604fd8c2a208de1753e6b1%7Ca6fa3b030a3c42588433a120dffcd348%7C0%7C0%7C638973846670618460%7CUnknown%7CTWFpbGZsb3d8eyJFbXB0eU1hcGkiOnRydWUsIlYiOiIwLjAuMDAwMCIsIlAiOiJXaW4zMiIsIkFOIjoiTWFpbCIsIldUIjoyfQ%3D%3D%7C0%7C%7C%7C&sdata=15MNFTb26CVltf2IeYz5mlP95jq86H%2B8HDtuJLK56Qk%3D&reserved=0)

2. Please include a complete copy of PLOS’ questionnaire on inclusivity in global research in your revised manuscript. Our policy for research in this area aims to improve transparency in the reporting of research performed outside of researchers’ own country or community. The policy applies to researchers who have travelled to a different country to conduct research, research with Indigenous populations or their lands, and research on cultural artefacts. The questionnaire can also be requested at the journal’s discretion for any other submissions, even if these conditions are not met.  Please find more information on the policy and a link to download a blank copy of the questionnaire here: <https://journals.plos.org/plosone/s/best-practices-in-research-reporting>. Please upload a completed version of your questionnaire as Supporting Information when you resubmit your manuscript.

The first (corresponding) and second authors are Ghanaian citizens. The first author is studying towards a PhD degree in Sport Science at a South African University. He visited South Africa during the proposal development and review, but all the field work for the study was conducted in Ghana and his local community. The inclusion of the second author (who served as PhD co-promotor) from Ghana was aimed at enhancing cultural relevancy during the data collection, analysis and write-up.

3. Your ethics statement should only appear in the Methods section of your manuscript. If your ethics statement is written in any section besides the Methods, please delete it from any other section.

The ethics statement appears in the Methods section, under “Procedure.” Two ethics committee’s approved the study, one in South Africa and another in Ghana.

4. In this instance it seems there may be acceptable restrictions in place that prevent the public sharing of your minimal data. However, in line with our goal of ensuring long-term data availability to all interested researchers, PLOS’ Data Policy states that authors cannot be the sole named individuals responsible for ensuring data access (<http://journals.plos.org/plosone/s/data-availability#loc-acceptable-data-sharing-methods>).

Data requests to a non-author institutional point of contact, such as a data access or ethics committee, helps guarantee long term stability and availability of data. Providing interested researchers with a durable point of contact ensures data will be accessible even if an author changes email addresses, institutions, or becomes unavailable to answer requests.

Before we proceed with your manuscript, please also provide non-author contact information (phone/email/hyperlink) for a data access committee, ethics committee, or other institutional body to which data requests may be sent. If no institutional body is available to respond to requests for your minimal data, please consider if there any institutional representatives who did not collaborate in the study, and are not listed as authors on the manuscript, who would be able to hold the data and respond to external requests for data access? If so, please provide their contact information (i.e., email address). Please also provide details on how you will ensure persistent or long-term data storage and availability.

The raw interview data generated in this study cannot be publicly shared due to ethical restrictions protecting participant confidentiality. Participants were elite professional footballers in Ghana whose detailed accounts contain sensitive information about career decisions and personal circumstances that could enable identification despite de-identification efforts. These restrictions were stipulated in the ethical approval from Stellenbosch University’s Research Ethics Committee for Social, Behavioural, and Educational Research (REC: SBE – 22265) and Kwame Nkrumah University of Science and Technology’s Committee on Human Research, Publications and Ethics (CHRPE/AP/304/21) and participant informed consent agreements. However, anonymised data underlying the specific findings of this study can be made available to qualified researchers upon reasonable request and subject to ethical review.

Data access requests should be directed to the institutional representative: **Dr. Ernest Adankwah, Department of Medical Laboratory Technology, Kwame Nkrumah University of Science and Technology, Kumasi, Ghana (email: eadankwah.chs@knust.edu.gh)**.

Requesters will be required to provide institutional credentials, research justification, and data use agreements ensuring ethical data handling. All digital data and backups will be stored on passworded protected devices. All printed copies of the data that captured the demographic details, sensitive information and signed consent sheet will be secured in a key-locked cupboard at the researcher’s personal office (Room 206) at Casely-Hayford Building, KNUST, Ghana. Additionally, all audio recordings and printed copies of data will be stored for five years and will be destroyed thereafter.

We believe this approach fully satisfies PLOS ONE data availability requirements while maintaining our ethical obligations to study participants. We remain committed to research transparency and are prepared to make anonymised data available to legitimate researchers through appropriate institutional channels.

5. If the reviewer comments include a recommendation to cite specific previously published works, please review and evaluate these publications to determine whether they are relevant and should be cited. There is no requirement to cite these works unless the editor has indicated otherwise.

There were no specific recommendations to cite specific published works.

**Comments to the Author**

1. Is the manuscript technically sound, and do the data support the conclusions?

The manuscript must describe a technically sound piece of scientific research with data that supports the conclusions. Experiments must have been conducted rigorously, with appropriate controls, replication, and sample sizes. The conclusions must be drawn appropriately based on the data presented.

Reviewer #1: Yes

Reviewer #2: Partly

2. Has the statistical analysis been performed appropriately and rigorously?

Reviewer #1: Yes

Reviewer #2: Yes

3. Have the authors made all data underlying the findings in their manuscript fully available?

The [PLOS Data policy](https://zaf01.safelinks.protection.outlook.com/?url=http%3A%2F%2Ftrack.editorialmanager.com%2FCL0%2Fhttp%3A%252F%252Fwww.plosone.org%252Fstatic%252Fpolicies.action%2523sharing%2F1%2F010f019a28db5b1b-094911dd-37d9-4638-8179-149c73c480e6-000000%2FpmteCZwMnkq2x7FkdLMlzSbmSGlBrbcGqa06MV6lL4Q%3D234&data=05%7C02%7C%7C4bb682144a604fd8c2a208de1753e6b1%7Ca6fa3b030a3c42588433a120dffcd348%7C0%7C0%7C638973846670631314%7CUnknown%7CTWFpbGZsb3d8eyJFbXB0eU1hcGkiOnRydWUsIlYiOiIwLjAuMDAwMCIsIlAiOiJXaW4zMiIsIkFOIjoiTWFpbCIsIldUIjoyfQ%3D%3D%7C0%7C%7C%7C&sdata=wIRfh9JJpzmA%2Bqowkatwq8D8%2Fqq%2BQUjg9JsHfDLBZ5k%3D&reserved=0) requires authors to make all data underlying the findings described in their manuscript fully available without restriction, with rare exception (please refer to the Data Availability Statement in the manuscript PDF file). The data should be provided as part of the manuscript or its supporting information, or deposited to a public repository. For example, in addition to summary statistics, the data points behind means, medians and variance measures should be available. If there are restrictions on publicly sharing data—e.g. participant privacy or use of data from a third party—those must be specified.

Reviewer #1: Yes

Reviewer #2: No

Please refer to the earlier comment regarding the availability of the transcripts. Please note that these transcripts have been cleaned up to protect participant information, by removing direct and indirect references to the participants and events that may disclose their identity. It must also be reiterated that these transcripts will be made available upon reasonable request only. Additionally, we provide extensive data within the manuscript, i.e., detailed demographic, themes, subthemes with in-vivo codes, and rich participant quotations throughout the results section, enabling researchers to assess our analytical process and evaluate transferability.

It is standard practice in Ghana and South Africa that hard copy and electronic versions of datasets are destroyed after five years post study.

4. Is the manuscript presented in an intelligible fashion and written in standard English?

PLOS ONE does not copyedit accepted manuscripts, so the language in submitted articles must be clear, correct, and unambiguous. Any typographical or grammatical errors should be corrected at revision, so please note any specific errors here.

Reviewer #1: Yes

Reviewer #2: Yes

5. Review Comments to the Author

Please use the space provided to explain your answers to the questions above. You may also include additional comments for the author, including concerns about dual publication, research ethics, or publication ethics. (Please upload your review as an attachment if it exceeds 20,000 characters)

**Reviewer #1 (Gyanesh Kumar Tiwari):**

Using a descriptive phenomenological design, the study aimed to investigate and characterize professional Ghanaian football players' viewpoints on the function of mental toughness in their decision-making. My observations are given below:

(1) The study title is okay.

(2) The abstract needs revision. Although the abstract is informative and well-structured, it is extremely detailed, particularly when it comes to reporting age statistics and extensive themes. Clarity and scholarly appeal would be enhanced by condensing results while highlighting novelty, psychological contribution, and wider implications.

We appreciate the reviewer’s constructive feedback on the abstract. We have revised the abstract to enhance clarity and scholarly appeal by:

1. Restructuring the narrative flow to progress logically from research gap and purpose, through methodological approach to key findings and implications, thereby enhancing readability.

2. We removed detailed demographic statistics while maintaining essential sample description.

3. Presenting the five decision-making domains more conceptually rather than listing each domain individually.

4. Foregrounding the study’s novelty in a virtually unexplored dimension of decision-making, specifically within the African football context.

5. Explicitly articulating the psychological contribution: that mental toughness extends beyond performance enhancement to function as a multidimensional decision-making resource, and

6. Strengthening practical implications by explicitly connecting findings to culturally tailored intervention design for resource-constrained contexts.

(3) The introduction also needs minor revision. A thorough overview of mental toughness, its conceptual difficulties, and its application to decision-making is given in the introduction. But because it is so long and contains so much detail, it loses focus. The shift from general literature to the specific research gap is slow, despite the fact that the cultural significance of football in Ghana is well-contextualized. Clarity would be increased by more precisely stating the gap, novelty, and particular goals. Conciseness is also weakened when conceptual problems are repeated. A clearer structure would improve readability and more successfully draw attention to the study's contribution.

We value the reviewer’s detailed observations on the introduction. We undertook a series of targeted revisions to enhance clarity, focus, and structural coherence. First, the introduction was streamlined by eliminating repetitive descriptions of conceptual issues surrounding mental toughness. This has allowed for a more concise presentation of the construct’s conceptual challenges without unnecessary reiteration.

Second, the progression from general literature on mental toughness to its specific relevance for decision-making was reorganized to create a clearer line of argument. The updated version now presents the broader conceptual terrain briefly before transitioning more directly into literature linking mental toughness with decision-making processes in the sports contexts.

Moreover, the cultural section was refined to ensure that the discussion on Ghanaian football more clearly supports the identification of the research gap. While maintaining its contextual importance, redundant detail was reduced to keep the narrative focused. This restructuring allowed for a more explicit articulation of the gap concerning how mental toughness is understood and navigated by Ghanaian footballers, which in turn strengthens the justification for the study.

Finally, the purpose of the study has been more clearly stated at the conclusion of the introduction to state the study’s novelty and goals. Collectively, these revisions improve readability, sharpen the line of argument, and enhance the contribution of the research in line with the reviewer’s suggestions.

(4) The method again needs minor amendments. The thorough materials and methods section provides a clear explanation of sampling, data collection, and analysis as well as a compelling philosophical defense of descriptive phenomenology. Trustworthiness tactics and ethical issues are thoroughly explained. However, the theoretical exposition in this section could be simplified to make it easier to read. Reliance on coaches as gatekeepers may introduce bias and should be more critically acknowledged, even though purposive sampling and data saturation are justified. Methodological rigor would be further improved by greater clarity regarding reflexivity procedures and limitations.

We thank the reviewer for this constructive feedback and appreciate the suggestions for improvement. We made substantive revisions to address these concerns.

1. We substantially revised the “Study design and philosophical orientation” section by consolidating and streamlining the phenomenological justification. The revised section has been reduced by approximately 33% (from ~ 234 to ~ 157 words) while maintaining the essential philosophical grounding necessary for readers to understand our methodological choices. We removed redundant theoretical language and restructured the narrative for easy readability.

2. The reviewer’s point about potential gatekeeper bias is well taken. Coach-mediated nominations can shape participant selection based on subjective perceptions or interpersonal dynamics. We, therefore, clearly acknowledged this risk and added mitigation strategies (cross-validation protocol that limits individual coach bias). We also explained why the mitigation strategies were deemed appropriate given the study’s phenomenological focus on lived experiences rather than prevalence estimation. This aligns with Patton’s (2015) guidance on elite-informant sampling and transparency in purposive recruitment.

3. The reviewer indicated that our original reflexivity description was insufficiently detailed. We have revised the reflexivity section in the Data Analysis subsection to provide concrete description of our reflexivity practices such as pre-research reflexivity meetings, reflective memos, ongoing bracketing, and continuous reflexivity meetings.

This revision transforms the vague statement “engaged in reflexivity” into concrete, transparent account of specific reflexivity strategies implemented throughout the research process. We believe these changes significantly strengthen the manuscript and address all concerns raised by the reviewer.

(5) The results may be improved. Rich, well-organized, and full of detailed participant quotes, the results section serves as a solid foundation for the thematic analysis. It is admirable how lower- and higher-order themes are integrated to highlight the complex aspects of mental toughness. However, the section lacks focus and clarity due to its excessive length and occasional repetition. Without sacrificing depth, some themes could be condensed into a shorter summary. Additionally, although tables are helpful, there is room for improvement in how they are incorporated into the story. This section would be strengthened by more critical interpretation in addition to descriptive reporting.

We appreciate the reviewer’s thoughtful and encouraging feedback. We have condensed the results section to improve focus and eliminate repetition, streamlined theme narratives while retaining phenomenological richness, improved the integration of tables, and incorporated clearer interpretive commentary alongside descriptive reporting. These revisions enhance clarity, coherence, and analytical depth without compromising participants lived experiences.

1. **Addressing Excessive Length and Improving Focus**

While we acknowledge the reviewer’s concern regarding length, we respectfully note that descriptive phenomenological research requires substantial space to honour participants’ lived experiences through their own voices – a fundamental methodological principle. Nonetheless, we have improved efficiency and focus throughout the section by eliminating redundant transitional phrases and streamlining narrative flow without removing participant quotes, which represent the essential experiential data of phenomenological inquiry. Specifically, we remove repetitive opening phrases; consolidated redundant explanatory text; reduced each theme section to progress more directly from phenomenon description to interpretive insight.

2**. Eliminating Repetition**

We carefully reviewed the entire results section to identify and eliminate repetitive content. Key revisions include:

- Removing redundant phrases by varying linguistic expression while maintaining conceptual clarity.
- Eliminated narrative text that unnecessarily duplicated information already presented in Table 1.
- Streamlined theme descriptions to avoid conceptual overlap while preserving distinctive features of each decision-making domain, and
- Ensured each participant quote serves a unique illustrative purpose without redundancy.

**3.** **Strengthening Table Integration**

- Adding explicit references to Table 1 in the “Summary of themes” section: “The overarching themes, lower-order themes, and their descriptions are detailed in Table 1…”
- Restructuring the narrative to leverage the table more effectively, allowing readers to reference comprehensive theme descriptions while the text focuses on interpretive insights.

This revision transforms the table from a supplementary element into an integral component of the results presentation, improving both efficiency and clarity.

**4. Adding Critical Interpretation Beyond Descriptive Reporting**

We substantially enhanced the interpretive depth throughout the results section while maintaining fidelity to descriptive phenomenological principles. Phenomenological research occupies a delicate methodological space; interpretation must remain grounded in participants’ lived experiences rather than imposing external frameworks prematurely (which belongs in the **Discussion**). However, we recognise the reviewer’s point that the results can articulate some interpretive context. We added brief interpretive commentaries that articulate why each theme matters for decision-making, linking the experiential descriptions to psychological processes (e.g., self-regulation, emotional control, leadership judgment). This provides analytical depth without drifting into theory-driven explanation, consistent with phenomenological boundaries.

Throughout the revisions, we ensured that participants’ voices remained central and that depth, nuance, and in vivo quotes were retained, consistent with descriptive phenomenology’s emphasis on rich experiential accounts.

(6) The discussion needs minor improvements. The conversation offers a comprehensive, culturally informed explanation of Ghanaian football players' mental toughness and emphasizes its multifaceted nature beyond performance improvement. Although the section is extremely descriptive and shows little critical engagement with contradictions or alternative explanations, the integration of existing literature is praiseworthy. A stronger theoretical contribution would result from a greater focus on how findings expand, improve, or contradict accepted theories. Furthermore, even though they were mentioned, the implications for applied practice could be developed more methodically. Clarity and scholarly impact would be improved by tightening the narrative.

We sincerely thank the reviewer for acknowledging the comprehensive and culturally informed nature of our discussion, as well as the integration of existing literature. We deeply appreciate the constructive feedback regarding opportunities to strengthen critical engagement, theoretical contribution, mechanistic elaboration, and applied implications. We revised the discussion and conclusion section to address each concern while maintaining fidelity to our descriptive phenomenological approach.

1. **Enhancing critical engagement with contradictions and alternative explanations**

We have substantially revised the discussion to include critical analysis of how our findings relate to existing theories, including where they challenge, extend, or refine established conceptualisation. Specifically, we have added:

- Critical examination of the perseverance-flexibility paradox: We addressed the apparent tension between mental toughness as perseverance and its adaptive flexibility in regulating different decision-making processes. This engages with and refines characterisations of mental toughness as exclusively perseverance-oriented.
- Engagement with Crust et al.’ caution: We address their caution regarding overreliance on mental toughness fostering rigid perseverance, demonstrating how our findings reveal that mental toughness incorporating contextual sensitivity mitigates these risks.
- Challenges to Western Individualistic frameworks: We overtly acknowledged that mental toughness theories developed in Western, individualistic contexts may underrepresent dimensions salient in collectivist, resource-constrained environments.

**2. Demonstrating how findings expand, improve, or contradict accepted theories**

We systematically revised each thematic discussion to explicitly articulate how our findings advance theoretical understanding. We carefully weaved theoretical understanding and mechanistic insights across themes. Collectively the discussion highlighted mental toughness as operating through interconnected process including executive control, emotional regulation, attentional control, and social navigation mechanisms. These additions positioned mental toughness as a decision-making resource rather than a performance limited attribute. We provided new references providing theoretical foundations for these mechanistic explanations.

**3. Developing applied implications more methodically**

We completely restructured this section to provide concrete and actionable recommendation with implications for talent development and sports practitioners all grounded in the data.

The mechanistic explanations we weaved into the discussion did not impose external frameworks but rather explicate psychological processes participants themselves described experiencing. The revised discussion maintains comprehensive coverage while presenting ideas more efficiently and coherently.

**Reviewer #2 (Anonymous)**

Comment 1:

The literature review in the introduction lacks logical coherence and clear conceptual groundwork. Specifically, in lines 30–37, the author discusses the importance and role of "psychological resilience" without first providing a clear definition or conceptual explanation, resulting in insufficient logical rigor. Additionally, the cited literature is predominantly from 2000–2015, lacking recent research findings. The author is advised to:1）Before discussing the significance of psychological resilience, first clearly define its concept; 2）Reorganize the logical sequence of the literature review to better align with the structure of scientific argumentation; 3）Incorporate relevant research findings from the past 5 years, particularly literature linking psychological resilience to decision-making processes; 4）Further demonstrate why "decision-making" is a key entry point for understanding psychological resilience.

We sincerely thank the reviewer for his thoughtful engagement with our manuscript. However, we would like to respectfully address a fundamental conceptual issue that affect the interpretation of this comment and subsequent feedback. This manuscript examines mental toughness, not psychological resilience. While these two constructs overlap as psychological resources, they are theoretically and empirically distinct in the sports psychology literature, with different conceptual foundations, measurement, approaches, and performance implications. Mental toughness refers to a constellation of cognitions, emotions, attitudes that support sustained goal pursuit and performance under pressure (Jones et al., 2002; Coulter et al., 2010; Gucciardi, 2017), whereas psychological resilience concerns process of recovery and adaptation from stress, trauma, or adversity (Sarkar & Fletcher, 2014). This distinction is critical; our manuscript did not discuss “psychological resilience” as the reviewer suggests (lines 30-37 of the original manuscript discuss mental toughness). All references to “psychological resilience” in the reviewer’s comments appear to reflect this conceptual confusion. We address this clarification while responding substantively to the reviewer’s remaining points.

**Conceptual grounding and definition**

The reviewer noted the introduction discusses psychological resilience “without first providing a clear definition or conceptual explanation.” We respectfully clarify that the introduction provides a definition of **mental toughness** in paragraph 1: “mental toughness has broadly been conceptualised as a constellation of values, attitudes, emotions, and cognitions that enable athletes to effectively pursue goals and sustain high-level performance despite facing adversity” (Coulter et al., 2010; Gucciardi et al., 2015). The definition, grounded in established literature, appears in the manuscript’s opening substantive paragraph, providing the conceptual foundation the reviewer suggests is missing. We acknowledged the reviewer’s concern regarding conceptual clarity and have further strengthened this definition in the revised introduction to ensure readers immediately grasp the construct under investigation and explicitly acknowledged ongoing theoretical debates in the field.

**Currency of literature and foundational references**

The reviewer observed that the cited literature is “predominantly from 2000-2015, lacking recent research findings.” We have updated the introduction, incorporating new references including a recent systematic review and theoretical development in mental toughness research. These additions enhance the manuscript’s currency and demonstrate ongoing scholarly engagement with emerging literature.

However, we respectfully note an important methodological principle, the predominance of references from 2000-2015 reflects the foundational, authoritative literature establishing menta toughness as a scholarly construct. These seminal works including Jones et al. (2002), Gucciardi et al. (2008, 2010, 2015), Crust et al. (2015), and Coulter et al. (2010) – represent the theoretical bedrock upon which contemporary mental toughness research is built. Dispensing with these foundational references would compromise the scholarly integrity and theoretical grounding of our work.

Moreover, we note that mental toughness research experienced substantial theoretical development during the 2000-2015 period, with landmark conceptual papers, measurement validation studies, and qualitative explorations establishing the field’s empirical and theoretical foundations. To contextualise our study without engaging this literature would represent a significant scholarly omission. Our approach in the revised introduction grounds the study in authoritative foundational work while incorporating recent developments ensuring theoretical continuity.

**Logical coherence and argumentative structure**

In response to the recommendation to strengthen logical coherence, we have carefully examined and further reorganised the introduction to follow a clearer argumentative sequence. The revised introduction follows a clear, logically coherent sequence:

1. Paragraph 1: Establishes mental toughness as an important psychological construct, acknowledging conceptual debates while providing working definition grounded in authoritative literature.

2. Paragraph 2: A synthesis of research linking mental toughness to decision-making under pressure, while acknowledging potential risks of overreliance.

3. Paragraph 3: Contextualises Ghanaian football’s cultural significance and the unique structural/cultural constraints shaping athletes’ experiences, establishing why this context matters theoretically, and

4. Paragraph 4: A clear articulation of the conceptual and empirical gap that motivates the present study, followed by the study aim.

This structure improves the logical progression from conceptual background to empirical rationale and clarifies the theoretical problem addressed by the study.

**Decision-making as key entry point**

The reviewer recommends further explanation of why decision-making is an important lens for examining “psychological resilience (we assume the reviewer means mental toughness given the manuscript’s actual focus). Given the study’s focus on mental toughness, we framed this point more precisely. The revised introduction explains that:

- We emphasise that mental toughness research has focused predominantly on performance outcomes rather than the psychological processes (like decision-making) through which performance is achieved.
- We cite evidence that mentally tough athletes tend to make more effective decisions under pressure, suggesting decision-making represents a critical mediating process through which mental toughness influence performance.
- We note that decision-making extends beyond in-game tactics to encompass career management, professional development, and life balance domains where mental toughness’s influence remains underexplored.
- We contextualise decision-making as particularly salient in resource-constrained environments where athletes face competing pressures that create complex decision-making demands.

This clarification strengthens the theoretical justification for examining mental toughness specifically through the lens of decision-making processes.

In summary, we have addressed the reviewer’s concerns by refining conceptual definitions, updating the literature with recent citations while retaining foundational authoritative references, reorganising the introduction for stronger logical coherence, and articulating more explicitly why decision-making provides a meaningful analytical entry point for understanding mental toughness. We also clarified the conceptual distinction between mental toughness and psychological resilience, which is essential for interpreting the manuscript’s theoretical grounding and contribution. We hope this clarification, alongside the substantive revisions, addresses the reviewer’s concerns while maintaining the study’s theoretical integrity and scholarly rigor.

Comment 2:

The research methodology section is relatively limited, relying solely on interviews for data collection and lacking supplementary methods that could enhance the validity and generalizability of the findings. A single method may result in insufficient explanatory power for conclusions and make it difficult to capture more direct relationships between psychological resilience and decision-making behaviors. The authors are advised to consider the following in future research designs:1） Incorporate quantitative scales, such as psychological resilience assessment tools (e.g., Mental Toughness Questionnaire 48, etc.); 2）Combine situational or behavioral tasks (e.g., simulated decision-making scenarios, competition strategy selection tasks) to obtain more objective indicators of decision-making performance; 3）Enhance the reliability and external validity of results through mixed-methods design. 4）It is recommended to provide specific evidence supporting data saturation.

We thank the reviewer for his/her detailed comments on the methodological approach. However, we respectfully note that several suggestions appear to arise from a misunderstanding of the study’s conceptual and methodological foundations. We clarify these issues while addressing the reviewer’s s recommendations.

**Conceptual clarification**

The study examines mental toughness rather than psychological resilience. The reviewer’s recommendation to incorporate “psychological resilience assessment tools” such as MTQ48 reflects a conceptual conflation. The MTQ48 is a measure of mental toughness, not resilience. This distinction is central to understanding the theoretical framing and methodological choices in the manuscript.

**Appropriateness of phenomenological methods**

The reviewer characterises our methodology as “relatively limited, relying solely on interviews” and suggests incorporating quantitative scales and behavioural tasks. We respectfully but firmly disagree with this characterisation, as it applies positivist epistemological criteria to interpretivist phenomenological research. Descriptive phenomenology is grounded in the philosophical tradition of Husserl and prioritises access to lived experiences through rich, first-person accounts (Moustakas, 1994; Giorgi, 2009). In this approach in-depth interviews are not a methodological limitation but rather the ‘gold standard’ for phenomenological inquiry, providing the depth and experiential richness necessary to highlight the essential structures of a phenomenon.

Moreover, the study did not depend on interviews alone. Data collection and validation included field notes, reflexive memos, analyst triangulation, and member checking. These procedures enhanced the credibility and ensured rigorous interpretation within a qualitative paradigm (Lincoln & Guba, 1985). The suggestion of introducing quantitative scales or prestructured behavioural tasks would fundamentally compromise the phenomenological stance by imposing predefined constructs on participants before their experiential meanings emerge, which could conflict with the principle of epoché central to descriptive phenomenology.

**Validity, generalisability, and qualitative trustworthiness**

The reviewer’s concerns regarding “validity and generalisability” reflect misapplication of quantitative quality criteria to qualitative research. Qualitative studies employ different but equally established quality standards including credibility, transferability, dependability, and confirmability. Our study demonstrates rigorous adherence to these standards through prolonged engagement, triangulation, member checking, and detailed description of analytical decisions. Phenomenological research does not aim for statistical generalisability. Instead it seeks depth and contextual insights that support transferability when readers evaluate the extent to which the findings may be applicable in their own contexts.

**Mixed-Methods Recommendations**

Mixed-methods designs are valuable for certain research aims but are not suitable for descriptive phenomenology. Descriptive phenomenology requires researchers to bracket preconceptions and theoretical assumptions to allow participants experiences guide the emergence of meaning, which would be compromised by administering quantitative measures before or during interviews. While mixed-methods research may be appropriate for future studies examining relationships between measured mental toughness and decision-making behaviours, it would not align with the philosophical commitments of the present design.

**Data saturation**

The reviewer requested “specific evidence supporting data saturation.” We provided this evidence: “Twenty players expressed interest to take part in the study, and by the 10th interview no new substantive insights emerged pertaining to the breath or specificity of themes indicating we have achieved data saturation [27]. We conducted two additional interviews to confirm this determination, resulting in a final sample of 12 participants” (lines 141-145). This approach is consistent with established qualitative research practices (Guest et al., 20006; O’Reilly & Parker, 2013) demonstrating justification for sample adequacy. Two confirmation interviews beyond the saturation point provide conservative validation that adequate data richness was achieved.

**Summary and future directions**

We affirm that the methodological approach reflects established phenomenological principles and aligns with the study’s aim of examining lived experience. We added clearer justification of our methodological decisions and expanded the limitations section to recommend mixed-methods designs for future work exploring measurable associations between mental toughness and decision-making performance.

Comment 3:

All participants in the current study were drawn from a sample of professional football players in Ghana, resulting in an overly concentrated sample source that may, to some extent, limit the external validity and generalizability of the conclusions. Mental resilience and decision-making processes are significantly influenced by cultural, socioeconomic backgrounds, and competitive systems. Relying solely on samples from a single country or cultural group may make it difficult to support conclusions with broad applicability. However, Ghanaian athletes as research subjects also hold certain value. For instance, their competitive cultural background, resource-constrained environment, and social pressure characteristics can provide an important supplement to mental resilience research that differs from samples in Europe and North America. Therefore, the authors are advised to:

1） Clearly articulate the rationale and limitations of sample selection in the paper, particularly the potential influence of Ghanaian athletes' cultural and environmental characteristics on mental resilience and decision-making;

We appreciate the reviewer for highlighting the importance of cultural, socioeconomic and competitive environments in shaping mental toughness and decision-making. We agree that these contextual factors can influence how psychological attributes manifest. We respectfully disagree with the characterisation of our Ghanaian-focused sample as a methodological limitation. Instead, we position this cultural specificity as a fundamental methodological strength aligned with contemporary cultural sport psychology principles. We acknowledge the reviewer’s continued use of the term “psychological resilience” and clarify that the study examines mental toughness.

**Rationale for a Ghanaian sample**

The decision to focus exclusively on professional footballers in Ghana was intentional and theoretically grounded. Contemporary cultural sport psychology emphasises the importance of context-specific research for understanding how psychological constructs develop and function within particular cultural settings. Cultural psychology researchers (e.g., Schinke & Hanrahan, 2009; Ryba et al., 2013; Blodgett et al., 2015) argue that culturally situated studies represent essential foundational work before any claims of universality can be made. From this perspective, a context-specific sample is not a methodological limitation but a methodological requirement for generating culturally informed insights.

The Ghanaian Premier League offered a rich sociocultural environment in which mental toughness is shaped by distinctive pressures, including resource-constraints, community expectations, and early career responsibilities as family providers. These characteristics provided an opportunity to explore dimensions of mental toughness that may not be readily observable in European or North American settings. The reviewer acknowledges this value, noting that Ghanaian athletes offer an important complement to existing research. This is precisely the contribution the study aims to make. Thus, rather than limiting our contribution, cultural specificity enables it.

**Addressing cultural influence and limitations**

We strengthened the manuscript to explicitly articulate the rationale for sampling Ghanaian players and the ways cultural context may influence mental toughness and decision-making. The introduction now positions Ghanaian football as culturally significant space shaped by socioeconomic pressures and collective expectations. The methods section clarifies why elite Ghana players were purposefully selected as information-rich cases capable of offering deep experiential insight. Throughout the discussion, we distinguish between potentially universal psychological mechanisms, such as executive control and emotional regulation, and culturally shaped manifestations, such as boundary setting in response to extended family obligations. This analytical approach directly addresses how cultural and environmental characteristics influence mental toughness and decision-making while acknowledging that some regulatory mechanisms may operate universally across contexts.

We also expand the limitation section to acknowledge that a culturally sample does not allow claims of broad applicability. Instead, the study offers transferability rather than generalisability, where we have provided thick description of context, participants, processes, and findings, enabling readers to assess transferability to their own contexts. We have incorporated in our future research directions that researchers employ cross-cultural studies to examine how mental toughness operates across different football environments, allowing researchers to identify which elements are universal and which reflect culturally specific demands.

**Summary**

We have extensively addressed the reviewer’s recommendations throughout the manuscript, providing clear rationale for sample selection, systematic analysis of cultural influences, distinction between universal and culturally specific findings, and acknowledgment of the need for future cross-cultural research. However, we respectfully maintain that our culturally focused approach represents rigorous, theoretically grounded cultural sport psychology research rather than a weakness that undermines the study’s contribution.

2）Consider including athletes from other countries or diverse cultural backgrounds in future research to enhance external validity.

We thank the reviewer for this valuable recommendation. We incorporated it directly into the revised “Limitation and future directions section”, where we overtly state that future studies should include athletes from multiple countries and cultural settings. This would allow systematic comparison across African and Western contexts and help distinguish universal elements of mental toughness from those shaped by specific sociocultural environments. We agree that cross-cultural research is an important next step for advancing theoretical understanding. At the same time, culturally grounded studies such as ours are necessary precursors to such comparisons. Cultural sport psychology scholars emphasise that deep contextual work within individual cultures should precede cross-cultural generalisation, as premature comparison risk imposing Western assumptions and overlooking culturally specific manifestations. Our study provides this foundational understanding for the Ghanaian context.

We appreciate the reviewer’s suggestion and have positioned it prominently in our Limitations and future direction section, signalling to the field that multi-national, culturally diverse samples examining mental toughness in decision-making would constitute important extensions of our foundational work.

3）If continuing to use only Ghanaian samples, strengthen the theoretical explanation of "context specificity" to avoid overgeneralizing conclusions.

We thank the reviewer for this invaluable comment which highlights the importance of clearly articulating context specificity to avoid unintended overgeneralisation. We have strengthened this aspect throughout the revised manuscript.

First, we ground our interpretation more distinctly in theoretical work demonstrating that mental toughness is shaped by cultural and environmental conditions. In particular, we draw directly on Gucciardi’s (2017) argument that manifestations of mental toughness are influenced by the demands, norms, and values of specific sporting contexts. This framework is used consistently in the ‘Discussion section’ to distinguish culturally shaped expressions of mental toughness from potentially universal psychological mechanisms.

Second, we systematically delineated this distinction across all thematic domains. For instance, when discussing long-term career decisions or general life management, we explain how Ghanaian athletes’ collectivist obligations and resource constraints may shape how mental toughness is expressed. In addition, we identified underlying processes such as executive control, emotional regulation, and attentional control that may operate across cultural contexts. This approach ensures our analysis remains grounded in the Ghanaian context while acknowledging areas where universal mechanisms may apply.

Third, we have been careful throughout the manuscript to avoid universal claims. Interpretations are intentionally framed as context-specific, using conditional language (“may represent,” “suggests”) when discussing applicability beyond Ghana. The revised conclusion section states that our findings reflect how mental toughness operates within collectivist, resource-limited settings, and that more research is needed to determine the extent to which these patterns emerge in other sport cultures.

Finally, the limitation and future directions section clearly addresses this issue by noting that while our culturally specific sample provides essential depth and contextual richness, cross-cultural research is necessary to determine how these findings translate to other environments.

We believe these revisions now provide a clear and theoretically grounded account of context specificity while ensuring that our conclusions remain appropriately positioned as culturally anchored and analytically precise rather than premature universal claims.

Comment 4: This paper lacks an in-depth exploration of the mechanisms through which psychological resilience influences decision-making. Current discussions on their relationship remain at the phenomenological level, lacking theoretical explanations and mechanistic elaboration, which limits the theoretical contribution of the study. The authors are advised to further investigate the potential psychological processes by which resilience may affect decision-making behaviors (e.g., stress regulation, attentional control, self-efficacy, cognitive flexibility, etc.) and introduce relevant theoretical frameworks (e.g., dual-process theory, self-regulation theory, etc.) for argumentation. Even if empirical validation is not immediately feasible, proposing a conceptual model or mechanistic hypothesis could enhance the theoretical depth and academic value of the paper.

We greatly appreciate this valuable feedback. The reviewer correctly noted that the initial discussion remained largely descriptive and did not sufficiently articulate the psychological mechanisms through which mental toughness shapes decision-making. We agree this limited the manuscript’s theoretical contribution and have substantially revised the discussion to address this concern.

**1. Acknowledgment of original limitation**

The reviewer correctly observed that the original discussion “remain[ed] at the phenomenological level lacking theoretical explanation and mechanistic elaboration.” This was indeed a weakness that limited the manuscript’s theoretical contribution. While descriptive phenomenology prioritises participants’ lived experiences, we recognise that descriptive phenomenology and theoretical elaboration need not be mutually exclusive. In the reviewed manuscript we have retained the integrity of participants’ accounts while incorporating accessible mechanistic exploration and theoretical framework throughout each thematic discussion.

**2. Mechanistic process now woven through the discussion**

We have integrated mechanistic explanations directly within each thematic domain, explicating the psychological processes through which mental toughness influences decision-making. This approach ensues that theoretical interpretations remain grounded in the participants’ description. The revised manuscript now elaborates on:

- **Executive control** supporting future-oriented decision-making and the capacity to delay immediate rewards, particularly in career decisions.
- **Emotional regulation** enabling athletes to redirect frustration and maintain composure in challenging performance moments.
- **Attentional control** supporting players’ ability to sustain task focus while inhibiting distracting provocations.
- **Cognitive flexibility** allowing athletes to balance perseverance with context-appropriate adaptation, and
- **Cognitive awareness** guiding strategic adjustments in leadership and interpersonal situations.

These mechanism enable mental toughness to function as a comprehensive decision-making resource rather than domain-specific performance enhancer. In effect, this integrated approach provided the comprehensive mechanistic framework the reviewer sought while maintaining natural flow within the thematic discussions rather than isolating theory from phenomenological findings.

**3. Theoretical frameworks added**

The reviewer specifically suggests introducing theoretical frameworks including dual-process process theory and self-regulation theory. The revised discussion now moves beyond describing that mental toughness influences decision-making and integrating these frameworks to explain how it does so. We highlight these throughout the discussion section:

- **Self-regulation theory:** We clearly connect findings to self-regulation frameworks when discussion professional development. We cite Duckworth et al.’s (2019) work on self-control and Toering and Jordet’s (2015) research on self-regulation in athletes, grounding mechanistic explanations in established self-regulation theory.
- **Dual-process framework:** We referenced dual-process operations in performance contexts. While we do not invoke dual-process theory by name (at it more commonly applies to system 1/system 2 reasoning), we describe the integrated operation of multiple regulatory processes consistent with dual-process thinking about decision-making.
- **Executive function theory:** We incorporate Diamond’s (2013) comprehensive framework on executive function throughout strategic career and professional development discussions, connecting mental toughness to core executive function components) that enable effective decision-making.
- **Emotion regulatory theory:** We draw on Gross’s (2015) process model of emotion regulation to explain how mental toughness enables productive channelling of emotional arousal rather than suppression or reactive expression, particularly in performance-based decision-making.
- **Cultural psychology frameworks:** We incorporated Markus and Kitayama’s (1991) seminal work on culture and self to theorise how collectivist cultural contexts shape mental toughness manifestations, particularly when discussing interpersonal and life management domains.

These revisions substantially deepen the manuscript’s theoretical contribution and provide a foundation for future empirical testing.

**Balancing phenomenological integrity and theoretical depth**

Importantly, we have added these mechanistic explanations in ways that explicate psychological processes participants themselves described experiencing, rather than imposing external theoretical frameworks disconnected from lived accounts. By weaving these explanations throughout each thematic discussions rather than separating them, we maintain the connection between lived experience and theoretical interpretation.

These revisions strengthen the manuscript’s theoretical depth while maintaining alignment with phenomenological principles.

This manuscript presents a topic with innovation and practical significance, particularly in integrating cultural context differences with psychological resilience—decision-making mechanisms. However, the current draft leans more toward doctoral dissertation outcomes and requires substantial improvements in theoretical construction, methodological transparency, logical coherence of results and discussion, as well as language refinement. A major revision is recommended before reconsideration.

**Concluding words from the authors**

We again want to thank the reviewers for their constructive feedback. We believe these major changes have improved our paper significantly and we look forward to the review of our revised manuscript.

Yours sincerely

The authors
